# The fluctuations of alpha power: Bimodalities, connectivity, and neural mass models

**DOI:** 10.1162/IMAG.a.64

**Published:** 2025-07-02

**Authors:** Jesús Cabrera-Álvarez, Alberto del Cerro-León, Blanca P. Carvajal, Martín Carrasco-Gómez, Christoffer G. Alexandersen, Ricardo Bruña, Fernando Maestú, Gianluca Susi

**Affiliations:** Department of Experimental Psychology, Universidad Complutense de Madrid, Madrid, Spain; Center for Cognitive and Computational Neuroscience, Universidad Complutense de Madrid, Madrid, Spain; Department of Bioengineering, University of Pennsylvania, Philadelphia, PA, United States; Department of Radiology, Rehabilitation and Physical Therapy, Universidad Complutense de Madrid, IdISSC, Madrid, Spain; Department of Structure of Matter, Thermal Physics and Electronics, Universidad Complutense de Madrid, Madrid, Spain

**Keywords:** alpha rhythm, MEG, resting state, DMN, Jansen-Rit, computational model

## Abstract

The alpha rhythm is a hallmark of electrophysiological resting-state brain activity, that serves as a biomarker in health and disease. Alpha power is far from uniform over time, exhibiting dynamic fluctuations. The likelihood of those power values can be captured by a decreasing exponential function, which in certain cases becomes bimodal. While alpha rhythm is usually evaluated through the averaged power spectra across entire recordings, its dynamic fluctuations have received less attention. In this study, we investigate the dynamic nature of alpha power, its relationship with functional connectivity (FC) within the default mode network (DMN), and the ability of the Jansen-Rit (JR) neural mass model to replicate these fluctuations. Using MRI and MEG data from 42 participants in resting state with eyes-closed and eyes-open, we evaluated the shape of the exponential distributions for alpha power fluctuations, and their relationship with other spectral variables as frequency, power, and the aperiodic exponent. Additionally, we assessed the temporal relationship between alpha power and FC using phase-based (ciPLV) and amplitude-based (cAEC) metrics. Finally, we employed diffusion-weighted MRI to construct brain network models incorporating JR neural masses to reproduce and characterize alpha fluctuations. Our results indicate that alpha power predominantly follows unimodal exponential distributions, with bimodalities associated to high-power in posterior regions. FC analyses revealed that ciPLV and cAEC were directly correlated with alpha power within the DMN in alpha and beta bands, whereas only theta-band ciPLV showed an inverse relationship with alpha power. JR model simulations suggested that post- supercritical fixed points better replicated alpha power fluctuations compared to limit cycle parameterizations and pre-saddle node fixed points. These results deepen our understanding of the dynamics of alpha rhythm and its intricate relationship with FC patterns, offering novel insights to refine biologically plausible brain simulations and advance computational models of neural dynamics.

## 1 Introduction

Alpha oscillations are a fundamental brain rhythm characterized by electrophysiological fluctuations at around 10 cycles per second. First identified by Hans Berger (Berger, 1929), these oscillations have emerged as a crucial biomarker both in health and disease (Babiloni et al., 2025; Ippolito et al., 2022; Ye et al., 2022; Zhang et al., 2024). Alpha activity manifests most prominently during periods of resting state with eyes closed (Barry et al., 2007; Ingram et al., 2024; Wan et al., 2018), but its power also appears modulated in task-related contexts such as working memory (Bonnefond & Jensen, 2012; Jensen, 2002; Palva & Palva, 2007; Tuladhar et al., 2007; Wianda & Ross, 2019) and attentional tasks (Foxe & Snyder, 2011; Kelly et al., 2006; Sauseng et al., 2005).

Alpha has been conceptualized both as an active state of cortical inhibition that avoids irrelevant stimuli during highly demanding cognitive tasks (Jensen, 2024) and as a passive state of cortical idling (Pfurtscheller et al., 1996). These two theories have been supported by multiple lines of evidence, including the observation of anti-correlations between BOLD signals and alpha power (Goldman et al., 2002; Liu et al., 2014; Ritter et al., 2008), the reductions in alpha amplitude within modality-specific regions during cognitive processing while increasing in task-irrelevant ones (Pfurtscheller, 1992; Worden et al., 2000), and the enhancements of alpha during states of focused relaxation, such as mindfulness and meditation (Katyal & Goldin, 2021; Lomas et al., 2015).

Alpha oscillations fluctuate in amplitude over time, exhibiting waxing and waning dynamics. The likelihood of power values can be captured by a decreasing exponential function (Freyer et al., 2009), indicating a higher prevalence of low-power states with an exponentially decreasing likelihood for higher power values. Intriguingly, in some cases, these distributions appear bimodal, suggesting two distinct modes of alpha activity—one with higher and another with lower amplitude (Freyer et al., 2012). Such bimodalities have been interpreted within the framework of neuronal criticality reflecting transitions between two states (Freyer et al., 2012).

At the network level, alpha has been associated with the activation of the default mode network (DMN) from a time-averaged perspective. Electrophysiological studies have reported increased functional connectivity (FC) in the DMN associated to higher alpha power (Clancy et al., 2021; Kim et al., 2023). Other authors propose a more nuanced relationship, suggesting a direct association only in the eyes-open resting state (Mo et al., 2013), and highlighting region-dependent variations—some areas exhibiting a positive correlation with alpha power, while others show an inverse relationship (Bowman et al., 2017). However, whether there is a relationship between the fluctuations of alpha power and network dynamics remains an open question. Given the anti-correlations observed in previous studies between alpha power and BOLD signals, we hypothesize to find also an indirect relationship between alpha power and the activation of the DMN, taking into account the spatial complexity and the temporal dynamics of both variables.

Finally, neural mass models, such as the Jansen-Rit (JR) (Jansen & Rit, 1995), are able to reproduce the waxing-waning fluctuations of alpha, in this case, through the interaction of biologically plausible neuronal inhibitory and excitatory subpopulations. However, these dynamics can be obtained with different types of parameterizations, including limit cycles regimes in which the model autonomously oscillates, and fixed points regimes in which the model behaves as a damped oscillator (Grimbert & Faugeras, 2006; Spiegler et al., 2010). The accuracy of each of those regimes to resemble the empirical electrophysiological fluctuations of power observed in M/EEG remains to be studied.

In this study, we aim to advance our understanding of alpha oscillations through a comprehensive characterization of power fluctuations across regions and considering two brain states (resting with eyes-open and eyes-closed). Using MEG data, we will analyze the likelihood distribution of power values through time, identifying regions exhibiting bimodal distributions, and quantifying their prevalence. Additionally, we will examine the relationship between alpha power fluctuations and FC in the DMN. Finally, we will evaluate the JR model to reproduce the shape of the empirically observed alpha fluctuations, discussing the theoretical implications of different model parameterizations, and commenting on the mechanisms underlying alpha fluctuations.

## 2 Materials and Methods

### 2.1 Dataset

MRI (T1 and DWI) scans and MEG recordings were acquired from 42 healthy participants (20 females) with mean age 70.76 (std 4.65) from a dataset owned by the Centre for Cognitive and Computational Neuroscience, UCM, Madrid.

MRI-T1 protocols were performed using a General Electric 1.5 tesla magnetic resonance scanner, using a high-resolution antenna and a homogenization PURE filter (fast spoiled gradient echo sequence, with parameters: repetition time/echo time/inversion time = 11.2/4.2/450 ms; flip angle = 12°; slice thickness = 1 mm, 256 × 256 matrix, and field of view = 256 mm).

DWI were acquired with a single-shot echo-planar imaging sequence with parameters: echo time/repetition time = 96.1/12,000 ms; NEX 3 for increasing the signal-to-noise ratio; slice thickness = 2.4 mm, 128 × 128 matrix, and field of view = 30.7 cm yielding an isotropic voxel of 2.4 mm. A total of 25 diffusion sampling directions were acquired with b-value = 900 s/mm^2^, plus one additional image with no diffusion sensitization (i.e., T2-weighted b0 images).

MEG recordings were acquired using an Elekta-Neuromag MEG system with 306 channels at 1000 Hz sampling frequency and using an online band-pass filtered between 0.1 and 330 Hz. The recordings consisted of a 3-minute resting-state session with eyes closed (rEC), followed by an additional 3-minute resting-state session with eyes open (rEO). During acquisition, participants remained seated inside the magnetically shielded room (VacuumSchmelze GmbH, Hanau, Germany). The head shape of the participants was acquired using a three-dimensional Fastrak digitizer (Polhemus, Colchester, Vermont), in addition to three fiducial points (nasion and left and right pre-auricular points) as landmarks. Four head position indicator (HPI) coils were placed on the participant’s scalp (two on the forehead and two on the mastoids) and their position was continuously monitored during the acquisition to allow for head position tracking. Last, two sets of bipolar electrodes were used to record eye-blinks and heart beats, respectively.

Informed consent was obtained prior to the recordings from all participants in accordance with the Declaration of Helsinki, and the study received ethical approval from the ethics committee of the Universidad Complutense de Madrid.

### 2.2 MEG processing

MEG recordings were preprocessed offine using the spatiotemporal signal space separation (tSSS) algorithm (Taulu & Hari, 2009), embedded in the Maxfilter software v2.2 (correlation limit of 0.9 and correlation window of 10 seconds), to eliminate magnetic noise and compensate for head movements during the recording. Continuous MEG data were preprocessed using the Fieldtrip toolbox (Oostenveld et al., 2011) in Matlab R2020b. An independent component analysis based on SOBI (Belouchrani et al., 1997) was applied to remove the eye-blink and cardiac signals from the data. Then, signals were visually inspected, and the remaining artefacts were identified and excluded from subsequent analysis.

Source reconstruction was performed using the Brainstorm toolbox (Tadel et al., 2011) in Matlab employing the minimum norm estimates (MNE) method (Dale & Sereno, 1993; Hämäläinen & Ilmoniemi, 1994) with *constrained dipoles* oriented normally to the cortical surface. This captures the typical orientation of the macrocolumns of pyramidal neurons (Tadel et al., 2019) and improves source localization accuracy (Dale & Sereno, 1993). Finally, source-space signals were averaged per region using the HCP parcellation scheme (Glasser et al., 2016) (see Supplementary Table S1).

### 2.3 Alpha fluctuations

We used the MNE package (Gramfort et al., 2014) in Python 3.9 to extract a time-frequency representation (TFR) of each signal using Morlet wavelets (7 cycles) over a frequency range from 2 to 40 Hz in steps of 0.25 Hz. We computed the power spectrum by averaging the TFRs across time. The resulting spectra were modeled using the *fooof* package for Python (Donoghue et al., 2020) in the frequency range between 2 and 40 Hz. The model separates the periodic and aperiodic components of the spectrum and estimates the offset, exponent, and knee of the latter. From the periodic components identified, we selected that with the highest power in alpha band (8–12 Hz) as the individual alpha frequency (IAF). In the case of no alpha peak detected by fooof, IAF was considered to be 10 Hz.

To evaluate the fluctuations of alpha power over time, we selected a frequency band of the TFR at IAF +/- 2 Hz. The TFR values were then averaged in band, and the results were scaled by a factor of 1e-19. Finally, we evaluated the distribution of power values by computing a histogram of 200 bins following Freyer et al. (2009). We evaluated the fit of two types of exponential functions to the distribution of powers: unimodal exponential $P(x)=\lambda e^{-\lambda x}$ , and bimodal exponential $P(x)=w\lambda_{1}e^{-\lambda_{1}x}+(1-w)\lambda_{2}e^{-\lambda_{2}x}$ . The goodness of fit was evaluated using the Bayesian Information Criteria (BIC): BIC=kln(n)−2ln(L) , where k is the number of parameters used (1 for unimodal and 3 for bimodal), n is the number of datapoints, and L represents the likelihood of the model. Lower BIC values implies better fit to the data.

### 2.4 Functional connectivity

The FC was estimated with two different metrics: the corrected imaginary part of the phase locking value (ciPLV; (Bruña et al., 2018)) for phase synchrony, and the corrected version of the amplitude envelope correlation with pairwise signals orthogonalization (cAEC; (Hipp et al., 2012; O’Neill et al., 2015)) for amplitude synchrony. These two metrics are based on the oscillatory model for brain activity (Buzsáki et al., 2012), and estimate functional connectivity from either phase (ciPLV) or amplitude (cAEC) synchronization. Although linked, these two aspects of the oscillatory activity are distinct (Siems & Siegel, 2020), and therefore we will evaluate them separately. Both measures correct for source leakage and volume conduction.

To compute these metrics, we epoched the signals using a sliding window approach with windows of 1 second—and additional padding of 1 second at each extreme of the window—and an overlap of 0.5 seconds. We filtered the epoched data in 4 different frequency bands: theta (4–8 Hz), alpha (8–12 Hz), beta (12–30 Hz), and gamma (30–45 Hz); and computed the Hilbert transform of the signals. The differences in the imaginary part of the Hilbert’s phases were used to compute ciPLV, while the orthogonalized envelopes of that transformation were correlated to get the cAEC. This process was performed per window, subject, and condition for each pair of regions in the DMN (see Supplementary Table S2).

### 2.5 Structural connectivity

DWI data were processed using DSI Studio (http://dsi-studio.labsolver.org). Firstly, the DWI data were rotated to align with the AC-PC line. The restricted diffusion was quantified using restricted diffusion imaging (Yeh et al., 2016). The diffusion data were reconstructed using generalized q-sampling imaging (Yeh et al., 2010) with a diffusion sampling length ratio of 1.25. The tensor metrics were calculated using DWI with a b-value lower than 1750 s/mm². A deterministic fiber tracking algorithm (Yeh et al., 2013) was used with augmented tracking strategies (Yeh, 2020) to improve reproducibility, using the whole brain as seeding region. The anisotropy threshold was randomly selected. The angular threshold was randomly selected from 15 degrees to 90 degrees. The step size was randomly selected from 0.5 voxels to 1.5 voxels. Tracks with lengths shorter than 10 or longer than 300 mm were discarded. A total of 1 million seeds were placed.

The HCPex v1.1 atlas (Glasser et al., 2016; Huang et al., 2021) was used as the volume parcellation atlas that complements the HCP atlas by including subcortical structures (see Supplementary Table S1). Finally, two structural connectivity (SC) matrices were constructed according to the count and average length of the streamlines connecting two regions.

### 2.6 Brain network model

SC matrices served as the skeleton for the brain network models (BNMs) implemented in The Virtual Brain (Sanz-Leon et al., 2015) where regional signals were simulated using JR neural mass models (NMMs) (Jansen & Rit, 1995). The JR is a biologically inspired model of a cortical column capable of reproducing alpha oscillations through a system of second-order differential equations (see Table 1 for a description of parameters):

**Table 1.** JR-BNM parameters used in simulations.

Parameter	Value	Unit	Description
H_e_	3.25	mV	Average excitatory synaptic gain
H_i_	22	mV	Average inhibitory synaptic gain
$\tau_{e}$	10	ms	Time Constant of excitatory PSP
$\tau_{i}$	20	ms	Time Constant of inhibitory PSP
C_pe_	135		Average synaptic contacts: pyramidals to excitatory interneurons
C_ep_	108		Average synaptic contacts: excitatory interneurons to pyramidals
C_pi_	33.75		Average synaptic contacts: pyramidals to inhibitory interneurons
C_ip_	33.75		Average synaptic contacts: inhibitory interneurons to pyramidals
e_0_	0.0025	ms^-1^	Half the maximum firing rate
r	0.56	mV^-1^	Slope of the presynaptic function at v0
v_0_	6	mV	Potential when half the maximum firing rate is achieved
p	variable	ms^-1^	Mean of random gaussian intrinsic noisy input
σ	variable	ms^-1^	Standard deviation of random gaussian intrinsic noisy input
g	variable		Coupling factor for inter-regional communication
s	3.9	m/s	Conduction speed for inter-regional communication

$${\dot{y}}_{0_{i}}=y_{3_{i}},$$ (1)

$${\dot{y}}_{1_{i}}=y_{4_{i}},$$ (2)

$${\dot{y}}_{2_{i}}=y_{5_{i}},$$ (3)

$$\tau_{e}{\dot{y}}_{3_{i}}=H_{e}S[y_{1_{i}}-y_{2_{i}}]-2y_{3_{i}}-\frac{1}{\tau_{e}}y_{0_{i}},$$ (4)

$$\tau_{e}{\dot{y}}_{4_{i}}=H_{e}\{I_{i}(t)+C_{ep}S[C_{ep}y_{0_{i}}]\}-2y_{4_{i}}-\frac{1}{\tau_{e}}y_{1_{i}},$$ (5)

$$\tau_{i}{\dot{y}}_{5_{i}}=H_{i}C_{ip}S[C_{pi}y_{0_{i}}]-2y_{5_{i}}-\frac{1}{\tau_{i}}y_{1_{i}},$$ (6)

where

$$\begin{array}{cc} S[v]=\frac{2e_{0}}{1+e^{r\left(v_{0}-v\right)}}, & \end{array}$$ (7)

$$I_{i}(t)=\eta_{i}(t)+g\sum_{j=1}^{n}w_{ji}S[y_{1_{j}}(t-d_{ji})-y_{2_{j}}(t-d_{ji})],$$ (8)

for i=1,…,N , where *N* is the number of simulated regions. The inter-regional communication introduces heterogeneity in terms of connection strength $w_{ji}$ and conduction delays $d_{ji}$ between nodes i and j where: $d_{ji}=\frac{L_{ji}}{s}$, with *L_ji_* being the length of the tract from node i to node j, and s representing the (global) conduction speed.

This model represents the electrophysiological activity (in voltage) of three subpopulations of neurons: pyramidal neurons ($y_{0}$), excitatory interneurons ($y_{1}$), and inhibitory interneurons ($y_{2}$) and their derivatives ($y_{3}$, $y_{4}$, and $y_{5}$, respectively). These subpopulations are interconnected through an average number of synaptic contacts (*C_ep_, C_ip_, C_pe,_* and *C_pi_*), and integrate external inputs from other cortical columns. The intra- and interregional communication is mediated through firing rates, which are determined by a sigmoidal function S converting voltage inputs into firing rates (eq. 7). The shape of the sigmoidal function is determined by its steepness r, its half-maximum $e_{0}$, and the voltage required to reach half-maximum $v_{0}$. The postsynaptic potential amplitudes (H_e_, H_i_), and time constants ($\tau_{e},\tau_{i}$) shape the oscillatory behaviour of subpopulations’ voltages.

The input ($I_{i}(t)$ ) represents two main drivers of activity in the NMMs: inter-regional communication and an intrinsic noisy input. The global coupling g scales the weight of the tracts connecting the brain regions of SC, as shown in (eq. 8). The intrinsic noisy input is defined as a local and independent Gaussian noise $\eta_{i}(t)∼N(p,\sigma )$ .

### 2.7 Simulations

All simulations were 60 seconds in length, from which we discarded 8 seconds to avoid initial transients. Sampling frequency was 1 kHz. Two types of simulations were carried out: single-node simulations and network simulations. For the latter, we downsampled the SC by averaging the 426 areas of the HCPex atlas into 66 regions defined in Supplementary Table S1.

To assess whether the simulated signals followed an exponential distribution, we computed the log(likelihood) of the simulated data given the exponential function derived from empirical recordings. To do this, we first normalized the empirical time-frequency representations (TFR(α)) within each region and then recomputed the parameters of the corresponding exponential distributions. This normalization ensured that both simulated and empirical TFR(α) were directly comparable on the same scale, allowing for a robust evaluation of their distributional properties.

### 2.8 Statistics

To assess statistical differences between conditions and brain regions in the parameterization of the exponential functions derived from empirical data, we conducted a series of Friedman’s tests as a robust alternative to one-way repeated-measures ANOVAs, given that homocedasticity assumption was not met.

We complemented goodness-of-fit (BIC) metrics, with an evaluation of the Kolmogorov-Smirnoff distance (KSD; (Hodges, 1958; Massey, 1951)) between the distributions of alpha power fluctuations and the theoretical distributions (e.g., unimodal exponential).

Additionally, we performed a set of Spearman’s correlation tests to relate the spectral characteristics of the signals (i.e., IAF, power, and aperiodic exponent) to the difference in BIC between unimodal and bimodal exponential functions, and to the value of KSD derived from the best performing model.

Finally, we evaluated the relationship between the FC and the fluctuations of alpha power by means of Spearman’s correlations. To this end, we downsampled in time the TFR(α) normalized per subject to match the time resolution of the FC analysis (windows of 1 second with steps of 0.5). This process generates a one dimensional array per region, which represents the level of alpha power across time. Then, for each pair of regions in the DMN, their average TFR(α) was correlated with the FC of the connection in time for each frequency band. Once with a TFR(α)-FC(band) correlation per metric and band, we averaged the correlations values per subject and performed an independent-samples t-test against a normal distribution centered at zero.

Corrections for multiple comparisons were performed with Bonferroni method.

## 3 Results

### 3.1 The distribution of alpha power fluctuations follows an exponential function

**Fig. 1.**
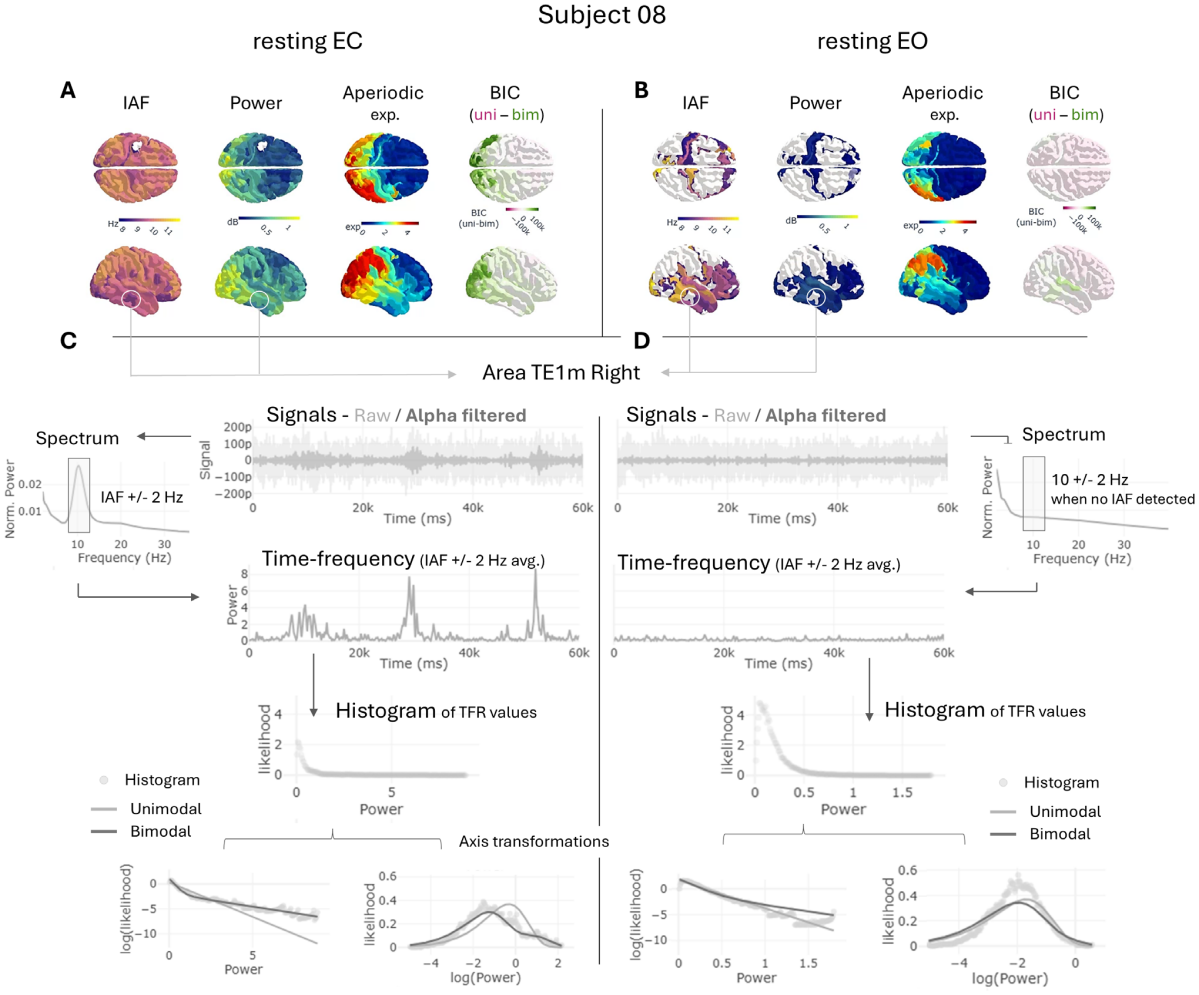
Pipeline of analysis for a sample subject (sub-08) in both rEC (A, C) and rEO (B, D) conditions. (A, B) Results of the spectral modeling including IAF, power, and the exponent of the aperiodic component. White regions in IAF and power represent non-detected alpha peaks. In these cases, we analyzed the 10 +/- 2 Hz frequency band. BIC column shows the difference in goodness of fit between the unimodal and bimodal exponential functions (positive values in green favor bimodality). (C, D) Alpha fluctuations analysis for one sample region (TEm1 R) both in rEC and rEO. First row, showing the signals (raw and filtered in alpha band); second, the spectra derived from the raw signal that is used to detect the IAF; third, TFR analysis in the IAF +/- 2 Hz frequency band averaged through frequencies to obtain a single array of alpha power in time; fourth, histogram with 200 equally sized bins using the data array of alpha power (each dot represents a bin); fifth, axis transformations of original exponential distributions helps to evaluate visually the presence of bimodalities. The fit of the unimodal and bimodal functions are shown in light and dark gray lines, respectively. Shared y-axis for rEC and rEO.

To evaluate alpha power fluctuations over time, we performed a time-frequency analysis of the signals, filtering them in the IAF +/- 2 Hz range, and averaging out frequencies to obtain an array of power values over time (see Fig. 1C, 1D). Then, we calculated the distribution of those power values by elaborating a histogram with 200 equally sized bins following Freyer et al. (2009). The resulting distributions followed exponential functions that in some cases were better represented by bimodal exponentials (see Fig. 1A, 1B - green regions in BIC). An example of these bimodalities can be appreciated in the axis transformations shown in Figure 1C, where the distribution of power for rEC was better captured by a bimodal exponential function [BIC = 181232, KSD(160000) = 0.08, p *<* 0.001] than by its unimodal counterpart [BIC = 216376, KSD(160000) = 0.17, p *<* 0.001].

In addition to that procedure, we evaluated the average spectral characteristics of the signals by computing their frequency spectra, modeling the aperiodic component, and measuring alpha frequency peak and power (Fig. 1A, 1B). Note that some regions do not show a significant alpha peak over the aperiodic component as modeled by fooof, specially in rEO (see Fig. 1B - blank regions in IAF and power). In those cases, the IAF used for the analysis was 10 +/- 2 Hz. Interestingly, the fluctuations of alpha in these regimes could also be accurately approximated by exponential distributions (see Fig. 1D).

### 3.2 Bimodal distributions are associated with high alpha power in posterior regions

**Fig. 2.**
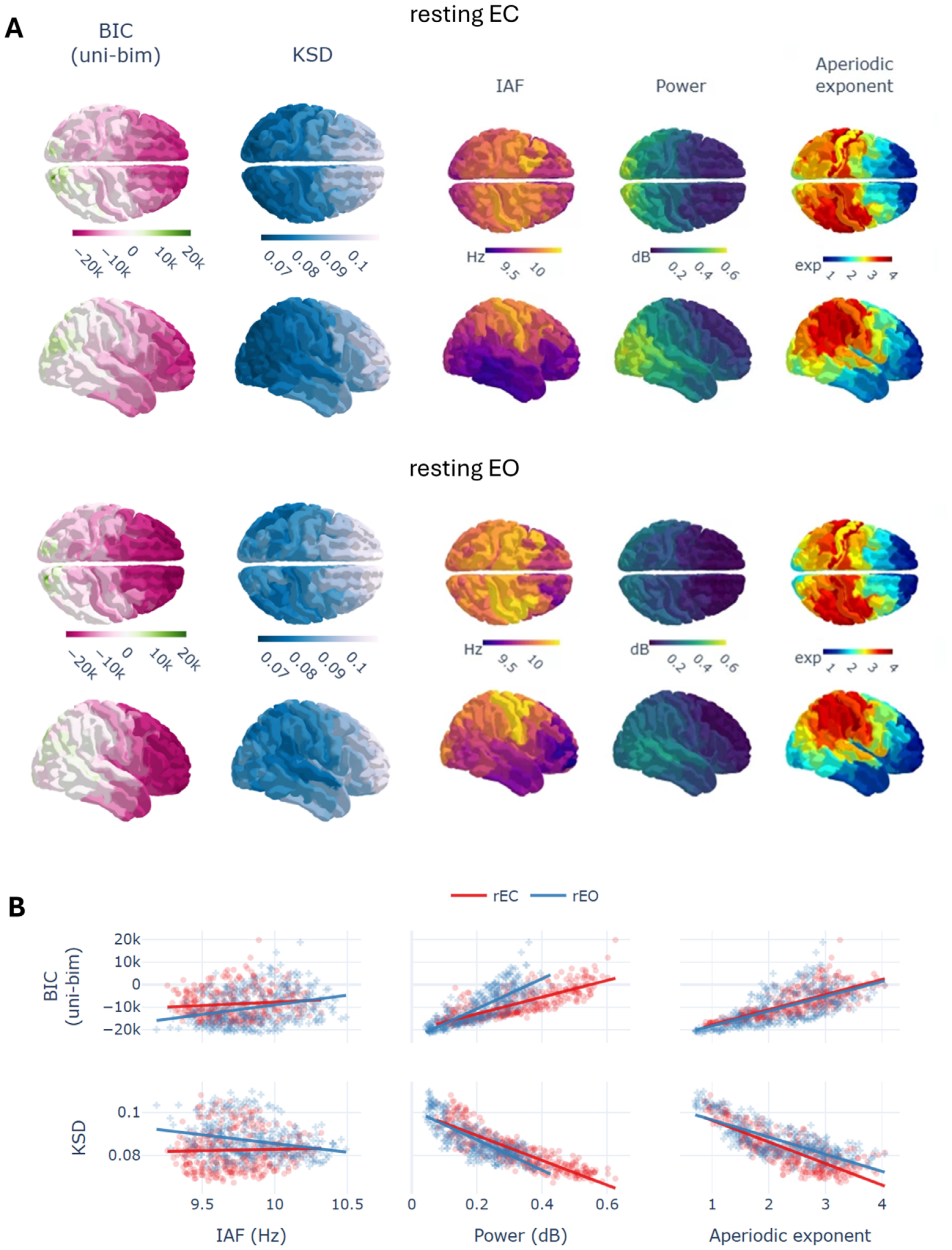
Regional characterization of the exponential modeling and spectral variables in rEC/rEO. Values were averaged through subjects. (A) Topological descriptions including the BIC difference between the unimodal and bimodal exponential fits (positive values in green favoring bimodality), the KSD value of the best fitted model per region (lower values in dark blue indicate better fit), and three spectral variables including IAF, alpha power, and aperiodic exponent. (B) Relationship between the exponential modeling variables and spectral ones. Note again that positive values in the BIC(uni-bim) favor bimodality and that lower KSD values imply better model fit.

Looking into the group averaged results, we observed that the bimodal distributions appeared most frequently over posterior regions, both in rEC and rEO (see Fig. 2A). We evaluated statistically this observation by performing two Friedman’s tests on the BIC differences between the unimodal and bimodal exponential fits using condition and space (i.e., brain region) as factors. We found significant differences for space [$\chi^{2}$ (359, 42) = 817.42, p-corr < 0.0001, W = 0.455] but not between conditions [$\chi^{2}$ (1, 42) = 0.857, p-corr = 1, W = 0.02]. Bimodalities appeared most often in posterior regions, but they were not always present, 11.90% of subjects showed no bimodality in rEC and 2.39% in rEO. In average, 15.42% (std 16.29%) of all brain regions were better characterized by bimodal exponentials, as indicated by lower BIC values compared to their unimodal counterparts. Frontal regions, in contrast, showed a clear tendency towards unimodality (see Fig. 2A).

Furthermore, we found strong correlations for the tendency towards bimodality and the spectral characteristics of the signal, specially for alpha power both in rEC [r(360) = 0.852, p-corr < 0.0001] and in rEO [r(360) = 0.923, p-corr < 0.0001] (see Fig. 2B - first row) but also for the aperiodic exponent in rEC [r(360) = 0.82, p-corr < 0.0001] and rEO [r(360) = 0.71, p-corr < 0.0001]. A slightly different picture was derived from the relationships between the spectral characteristics and the KSD values of the (best performing) exponential model. In this case, we observed inverse correlations for power [rEC: r(360) = -0.89, p-corr < 0.0001; rEO: r(360) = -0.78, p-corr < 0.0001], the aperiodic exponent [rEC: r(360) = -0.78, p-corr < 0.0001; rEO: r(360) = -0.73, p-corr < 0.0001], and with smaller effect size for IAF in rEC [r(360) = -0.23, p-corr < 0.0001] (see Fig. 2B - last row).

Interestingly, when comparing directly spectral characteristics between rEC and rEO, we only found a significant difference between conditions for power [$\chi^{2}$ (1, 42) = 13.71, p-corr < 0.002, W = 0.326] with higher alpha for rEC, but not for aperiodic exponent [$\chi^{2}$ (1, 42) = 0.095, p-corr = 1, W = 0.0022] and IAF [$\chi^{2}$ (1, 42) = 1.523, p-corr = 1, W = 0.036] (see Supplementary Fig. S1).

Finally, all KSD tests performed were statistically significant, which means that the distributions of alpha power fluctuations were different from the theoretical distributions. This could be related to the statistical power derived from the large sample sizes of the distributions compared (180000 datapoints). In the Supplementary Figure S2, we included additional KDS tests for other theoretical distributions that show better results for other exponential functions such as gamma and weibull but, especially, for the lognormal distribution. Again, all of them showed statistically significant differences.

In summary, these results show that higher alpha power as measured in the spectrum is related to bimodalities in the distribution of alpha fluctuations. Also, that the higher the power the better the fit to a theoretical exponential function, given by the decrease in distance in the KSD tests.

### 3.3 The FC of the DMN is directly related to the amplitude of alpha oscillations

In this section, we studied how the fluctuations of alpha power are related to the activity of the DMN. We expected to find differences in network activation depending on the levels of alpha, and more specifically, to find a negative relationship between the power of alpha and the FC in the DMN. We evaluated the FC within this network using two complementary measures: ciPLV to evaluate phase synchrony and cAEC to evaluate amplitude synchrony. Then, we correlated FC values with the alpha power in time for each pair of DMN regions. Finally, we averaged the correlation values per subject within the network, and made a group test against zero to evaluate for significant relationships between the variables.

**Fig. 3.**
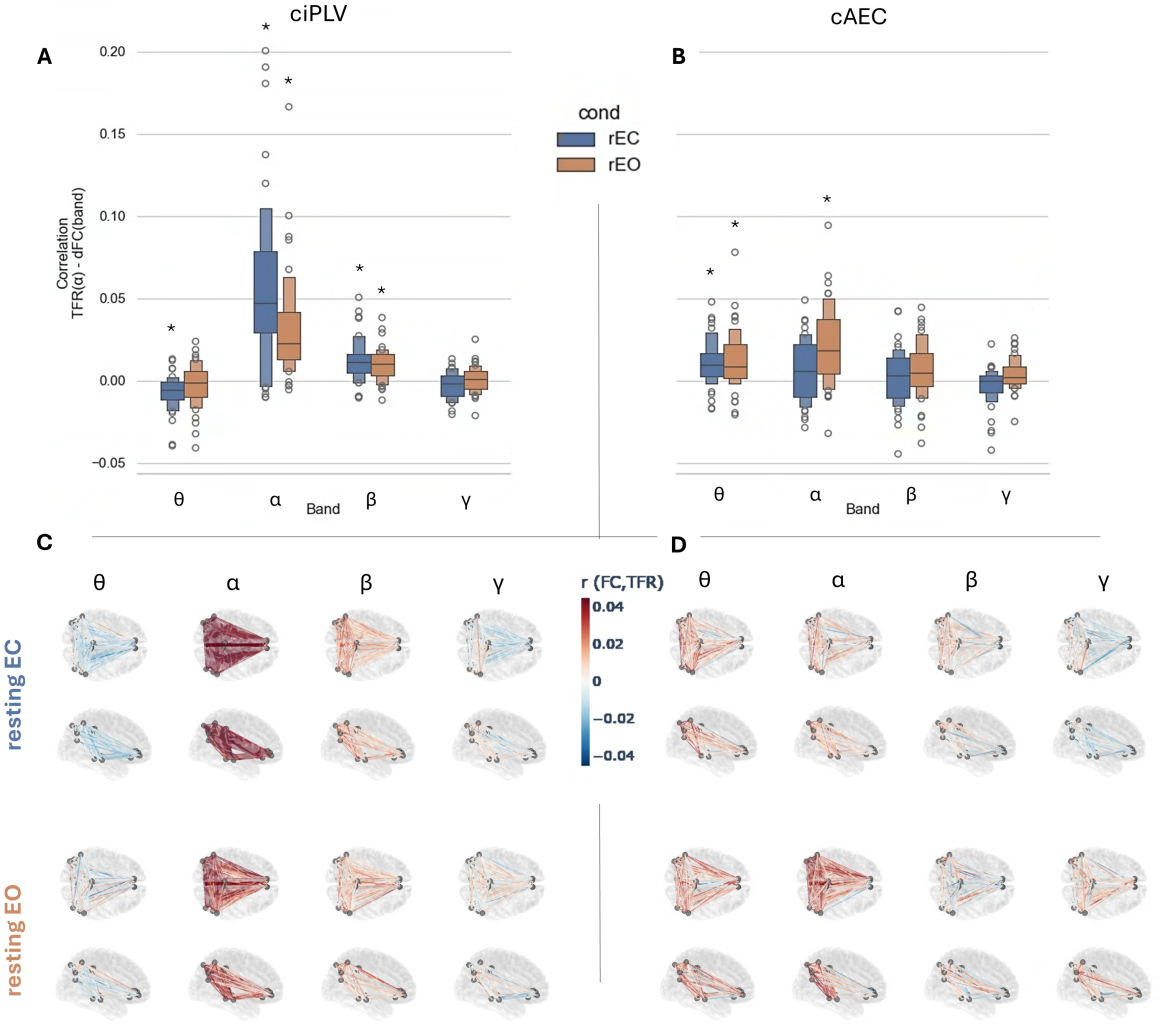
(A, B) Correlations between alpha power and FC in the DMN computed per frequency band and averaging across connections (each datapoint represent one subject), differentiating by FC metric (ciPLV/cAEC in panels A/B, respectively) and condition (rEC in blue and rEO in orange). Both panels share y-axis. (C, D) Correlations between alpha power and FC per band averaging out subjects to show each connection in the DMN differentiating by FC metric (ciPLV/cAEC in panels C/D, respectively). In color, the direction of the correlation with positive in red, negative in blue. (*) corresponds to statistical significance after correction for multiple comparisons, with p-corr < 0.01.

Both metrics yielded complementary insights on the relationship between alpha power fluctuations and FC (Fig. 3). With ciPLV, we observed a consistent direct relationship between alpha power and FC in alpha band [rEC: r(avg) = 0.057, T(39) = 6.95, p-corr < 0.001, Cohen’s d = 1.09; rEO: r(avg) = 0.032, T(39) = 6.06, p-corr < 0.001, Cohen’s d = 0.96] and beta [rEC: r(avg) = 0.012, T(39) = 5.72, p-corr < 0.001, Cohen’s d = 0.90; rEO: r(avg) = 0.0097, T(39) = 5.86, p-corr < 0.001, Cohen’s d = 0.92] bands. Interestingly, for ciPLV in theta band we found a tendency towards negative relationships that resulted significant for rEC [r(avg) = -0.007, T(39) = -4.12, p-corr < 0.005, Cohen’s d = 0.65]. With cAEC, FC resulted in a direct and significant relationship for alpha FC in rEO [r(avg) = 0.022, T(39) = 5.60, p-corr < 0.001, Cohen’s d = 0.88] but not in rEC[r(avg) = 0.006, T(39) = 2.24, p-corr = 0.48]. In this case, FC in theta band showed direct and significant relationships [rEC: r(avg) = 0.011, T(39) = 4.70, p-corr < 0.001, Cohen’s d = 0.74; rEO: r(avg) = 0.013, T(39) = 4.30, p-corr < 0.005, Cohen’s d = 0.68] while beta and gamma did not.

We performed an additional set of analyses focusing on complementary brain networks such as the visual, sensorimotor and attention networks (see Supplementary Fig. S3). Results showed similar associations between FC and alpha power as those described for the DMN. Interestingly, the most intense associations -even larger than those of the DMN- were observed for the visual [ciPLV(α)-rEC: r(avg) = 0.078, T(39) = 7.9, p-corr *<* 0.001, Cohen’s d = 1.25] and the dorsal attention networks [ciPLV(α)-rEC: r(avg) = 0.076, T(39) = 8.82, p-corr < 0.001, Cohen’s d = 1.39]. The sensorimotor network showed less intense but more consistent associations [e.g., ciPLV(α)-rEC: r(avg) = 0.04, T(39) = 8.98, p-corr *<* 0.001, Cohen’s d = 1.42]. Finally, only the visual network showed an inverse relationship for ciPLV in theta band with rEC [r(avg) = -0.0096, T(39) = -5.16, p-corr < 0.001, Cohen’s d = 0.815], mimicking the effect observed for the DMN.

### 3.4 The fixed points in Jansen-Rit reproduce better alpha power fluctuations

The JR model is widely used to simulate electrophysiological alpha oscillations. Here, we evaluate to what extent and under which parameterizations the JR reproduces the empirically observed power fluctuations. We followed a constructive approach by simulating first a single node and then whole-brain networks to explore the parameter spaces and model performance.

**Fig. 4.**
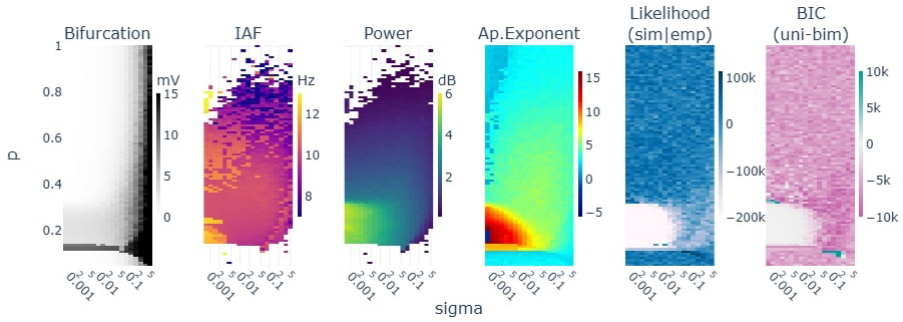
Parameter space explorations for a single node varying the mean intrinsic input (p) and standard deviation (sigma). In columns, 1) the bifurcation as the signal’s max-min voltage, 2) the alpha frequency peak, 3) the peak’s power and 4) aperiodic exponent as modeled with fooof toolbox, 5) the log(likelihood) of the unimodal exponential fit, and 6) the BIC difference between the unimodal and bimodal exponentials: the higher favors unimodal distributions. Blank values in columns 2) and 3) correspond to undetected alpha peaks by fooof modeling.

**Fig. 5.**
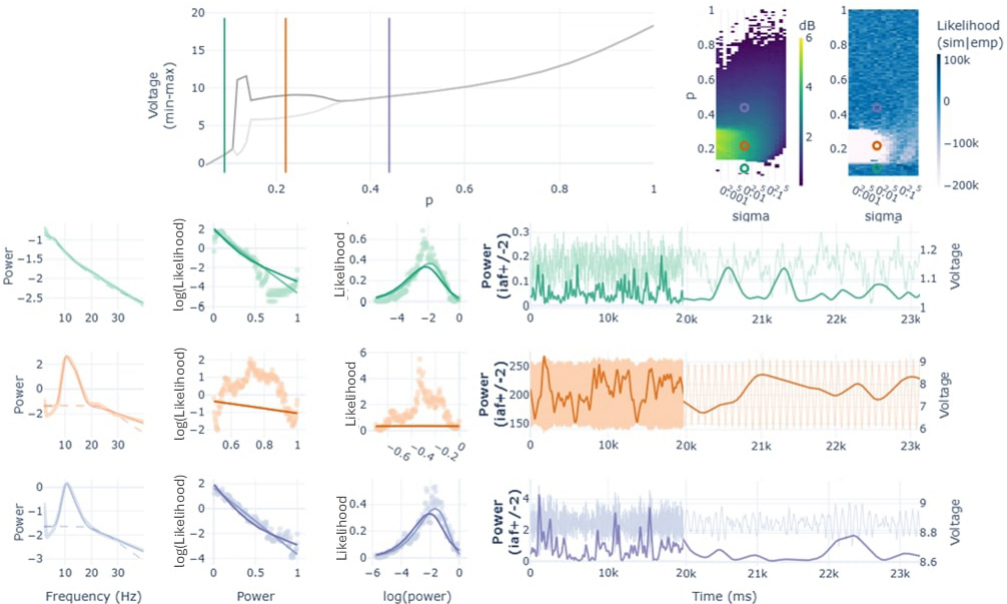
Three samples of the single-node simulations with p = [0.09, 0.22, 0.44] and sigma = 0.01. First row shows the bifurcation and two parameter spaces as reference for the picked simulations (in colored vertical lines and open circles). The following rows show: 1) the power spectrum calculated from the simulated signals (thick line), the modeled spectra (thin line), and the modeled aperiodic component (dashed line); 2) the exponential function in two different coordinate spaces (i.e., Linear-Log and Log-Linear) with the scatter representing the histogram of the simulated TFR(α) and the lines representing both the unimodal (light color) and the bimodal exponential models (dark color); 3) the raw signal (light color) and the TFR(α) values (dark color) in two different timeframes: 0–20 seconds for a broader overview, and 20–23 seconds for finer detail.

The results of single-node simulations showed better fits to the average empirical exponential function when the model was operating in a regime of attracting fixed points (steady states) (see Fig. 4 - 5th column) both before the saddle node (parameter p<0.11 ) and after the supercritical (parameter p>0.33 ) bifurcations of the JR. Between these two critical points, the JR exhibits limit cycle behaviours, with a worse fit to the empirical exponential. Also, increasing the levels of noise (i.e., σ) in the limit cycle regimes increased the goodness of fit.

We extracted a subset of samples from the parameter spaces to get a better grasp of the simulation results. In Figure 5, we show the signals, TFR, averaged spectrum and exponential modeling results for three different parameter combinations (i.e., sigma = 0.01, p = [0.09, 0.22, 0.44]). The simulation performed within the regime of stable limit cycle (e.g. p = 0.22) resulted in less biologically plausible alpha rhythm (Likelihood(sim—emp) = -289256.28) with a constant high power and little fluctuations, compared to the regimes with stable fixed points (Likelihood(sim—emp) = [38316.90, 22853.69]; Fig. 5 - see the bifurcation diagram as reference).

Regarding the fixed point states, we noticed that the fixed point regimes after the supercritical bifurcation (parameter p>0.33 ) show an alpha peak over the aperiodic component (see Fig. 5 - first column, last row) in contrast to the fixed point regime before the first saddle node bifurcation in which an alpha peak does not appear (parameter p < 0.11; see Fig. 5 - first column, green trace).

Regarding bimodalities, we expected them to appear at the critical points where the system could theoretically transition between different states of alpha. In this line, we found two sets of simulations favoring bimodal exponentials: one related to the first saddle node bifurcation (p ≈ 0.1025) and another related to the supercritical one (p ≈ 0.33). At the supercritical bifurcation with low noise (Fig. 4 - last column [p ≈ 0.33, sigma ≈ 0.005], in blue) we found a bimodal behavior that was artificially produced by the slowly decaying initial transient that overcome the discarded initial 8 seconds of simulation (see Supplementary Figs. S4 and S5). In contrast, at the saddle node bifurcation with higher noise (Fig. 4 - last column [p ≈ 0.1025, sigma ≈ 0.1]) an actual switching behavior was found between the fixed point and the limit cycle regimes of the JR (see Supplementary Fig. S6). The switching consisted in events/periods of high voltage alpha fluctuations intertwined with periods of a low voltage noisy signal characteristic of the fixed points.

These results suggest that the fixed point regimes reproduce the empirically-observed alpha power fluctuations, and that the model does not show biologically plausible bimodalities in the explored parameter space.

### 3.5 Theory and simulations favor post-supercritical fixed points to reproduce electrophysiological alpha oscillations

We simulated the whole-brain network to evaluate how the interactions between nodes could affect the fluctuation dynamics. We fixed the noise level (i.e., sigma = 0.001) and explored both the coupling factor (i.e., g) and the mean intrinsic input (i.e., p) parameters. The simulations were performed using the averaged SC of the sample of subjects.

**Fig. 6.**
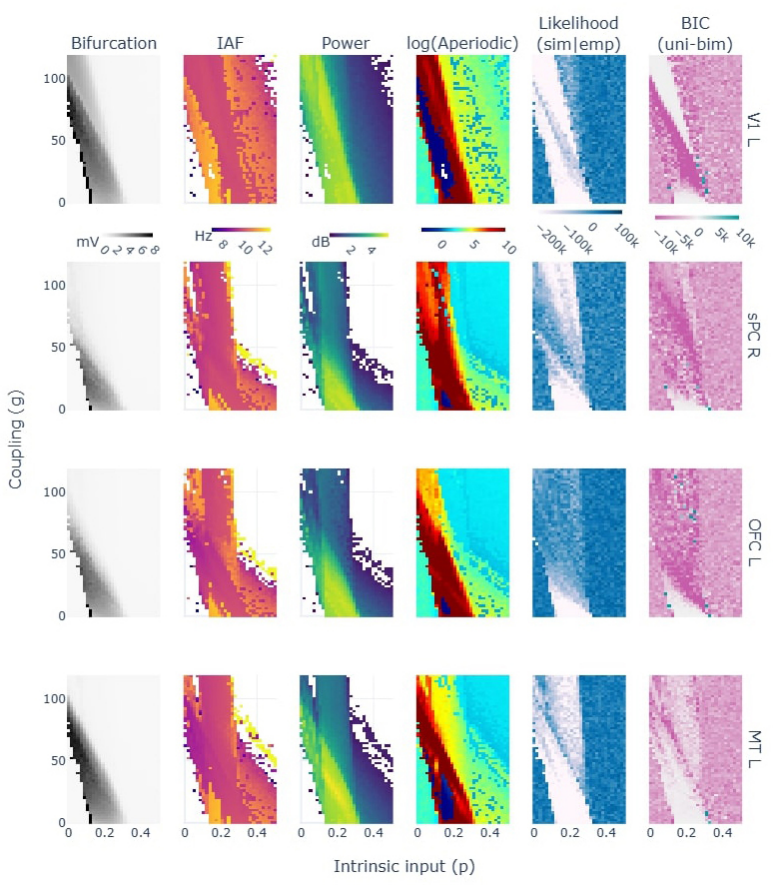
Parameter space explorations for network simulations with a fixed sigma = 0.001 and varying p and g. In columns, 1) the bifurcation as the signal’s max-min voltage, 2) the alpha frequency peak, 3) the peak’s power and 4) aperiodic exponent as modeled with fooof toolbox, 5) the BIC of the unimodal exponential fit, and 6) the BIC difference between the unimodal and bimodal exponentials: the higher favors unimodal distributions. Not shown values in columns 2 and 3 correspond to undetected alpha peaks by fooof modeling. Each row corresponds to analysis from simulated signals in different brain regions. Abbreviations: Orbito-Frontal Cortex (OFC), Medial Temporal Cortex (MT), Superior Parietal Cortex (sPC), Primary Visual Cortex (V1).

In the resulting parameter spaces, we found a bifurcation with a diagonal shape (see Fig. 6), which is not surprising given the roles of g and p as bifurcation parameters in the model. Apart from this difference, the spectral results and the exponential fits followed similar lines of single-node simulations. The worst fit of the unimodal exponential was found within the limit cycle of the JR. Before and after the limit cycle, the fluctuations of alpha power adjusted better to an exponential function. Although higher rates of bimodality might have been expected in these network simulations due to node interactions, they were, in fact, rare and sparsely distributed across the two main bifurcations of the model. In general, the model generated alpha fluctuations that were better captured by unimodal exponential distributions.

Similarly to single-node analysis, alpha peaks were found in post-supercritical bifurcation fixed points, while no alpha peak was detected in the pre- saddle node ones (see Fig. 6 - Power column; and Supplementary Fig. S7). Also, the spectral alpha power tends to decay as the node progresses further into the post-supercritical bifurcation regimes.

These results suggest that the fixed points in the post-supercritical bifurcation reproduce better electrophysiological alpha fluctuations than the typically used limit cycle operation state. We argue this given that: 1) fixed points reproduce better an exponential function of alpha fluctuations than the limit cycles, 2) the pre-saddle node fixed points do not show alpha activity in contrast to post- supercritical fixed points, and 3) the progressive reduction in alpha power through the post- supercritical bifurcation matches the theory of alpha claiming that it reflects inhibition / an idling state, in which case, the increase of input to the region would generate a reduction in alpha power.

## 4 Discussion

In this study, we have characterized the temporal fluctuations of alpha power, modeling them as exponential distributions and examining topological differences between rEC and rEO conditions. Additionally, we investigated the relationship between alpha power fluctuations and FC within the DMN, and assessed the performance of the JR neural mass model to accurately reproduce the shape of the empirically observed fluctuations, both in isolation and as part of a broader brain network model.

Our findings corroborate prior research suggesting exponential distributions of alpha power fluctuations (Evertz et al., 2022; Freyer et al., 2009), with bimodal patterns linked to high-power states in posterior regions. Although those previous studies focused on younger populations (ages ranging from 21–31 years), bimodality also appeared in our older sample, though interestingly found less frequent and less pronounced. In this sense, the alpha brain dynamics observed could be present across the lifespan, with a dampening of intensity in older adults due to neurophysiological changes in the aging process.

In their work, Freyer et al. (2009, 2011) proposed a mechanistic explanation for this phenomenon of bimodality, suggesting that the thalamocortical system operates in a subcritical Hopf bifurcation regime that allows it to switch between two states of alpha -low and high alpha amplitude-. Besides this hypothesis, we believe that those two states of alpha could reflect functionally different modes of the resting state. Resting state is a complex and dynamic condition in which participants are instructed to remain seated and relaxed, without engaging in any specific task. However, spontaneous mental activities, such as mind-wandering, episodic memory recall, and internal thought processing, may occur involuntarily during these periods (Christoff et al., 2016; Raichle et al., 2001; Smallwood & Schooler, 2015). The switching over time between an internal focus of attention—as in the case of mind wandering—and an external one might contribute to the differentiation of two modes of alpha (Fox et al., 2015; Sadaghiani & Kleinschmidt, 2016). This switching behavior could also be affected by age as shown in cross-modal attention tasks (Callaghan et al., 2017; Schils et al., 2024). Finally, from the technical point of view, we believe that volume conduction and source leakage could have intensified the observed bimodal effects by mixing signals from different sources. The use of MEG data in this study with higher spatial resolution and the use of source reconstruction methods might have reduced the prominence of this phenomenon.

The simulations performed with the Jansen-Rit (JR) model in this study failed to reproduce biologically plausible bimodal dynamics under the specific parameterization employed. However, alternative parameterizations of the JR model change significantly its behaviour such as showing subcritical bifurcations (Spiegler et al., 2010) that may offer a promising framework for capturing such bimodal behavior. In line with the hypothesis of dual resting-state modes, we suggest that reproducing this phenomenon may require the integration of additional biological mechanisms into the network models, particularly those related to neuromodulation, attention and perception. These enhancements could establish a neurophysiological basis for spontaneous transitions between distinct resting states, without requiring modifications to the neural mass model itself.

Regarding the relationship between alpha power fluctuations and dynamical FC, both phase (ciPLV) and amplitude (cAEC) metrics of FC showed direct relationships *within* the DMN. For this matter, *where* each aspect is evaluated becomes key. For instance, some studies have related the activation of the DMN with alpha power *in the visual cortex*, showing also direct relationships (Clancy et al., 2021; Kim et al., 2023; Mo et al., 2013). In our case, we have evaluated both alpha power and FC *within* the regions of the DMN. That is why, we hypothesized to find an inverse relationship (Bowman et al., 2017; Scheeringa et al., 2012), based on multimodal fMRI-EEG studies showing that higher alpha power is associated to lower BOLD signal (Goldman et al., 2002; Laufs et al., 2003; Pang & Robinson, 2018). Only theta band ciPLV in rEC resulted in significant negative relationships. Similar results were found when evaluating other complementary networks such as visual, sensorimotor, and attention networks always evaluating FC and alpha power *within* the network. These findings raise important questions regarding the intricate relationship between different neuroimaging modalities and their respective FC measures, emphasizing the need for integrative approaches to bridge gaps between electrophysiological and hemodynamic signals (Logothetis, 2008; Mulert, 2013; Ritter & Villringer, 2006). If high alpha power—associated with reduced BOLD signal— suggests a state of inhibition or idling (Jensen, 2024; Pfurtscheller et al., 1996), in the DMN it would suggest a state of disengagement from mind-wandering. How could electrophysiological measures of FC within that network concurrently indicate an increase? What would FC mean in that context? Future research should further investigate the mechanisms underlying these interactions and their implications for cognitive and neural processing.

It is important to add that age-related neurophysiological changes may be influencing these alpha dynamics or FC patterns. Literature describes a decrease in functional connectivity associated with aging, as measured by both EEG (Chow et al., 2022; Dadfar et al., 2025) and fMRI (Toussaint et al., 2014). In parallel, our group recently observed that age-related changes in alpha power in occipital regions exhibit a cubic function, with an initial increase in power during the first decades of life followed by a slight decrease and a subsequent increase from middle age onwards (Doval et al., 2024). Such dynamics may account for slight changes in the occipital alpha power and connectivity relationship between adolescence and middle age with respect to that observed in this study. Further research is needed to determine whether these trends are observed in other age groups.

On the computational side of this work, our results suggest the use of the JR’s fixed points—instead of its limit cycles or critical points—to simulate electrophysiological dynamics. Furthermore, we found a better performance for the post-supercritical fixed points over the pre-saddle node ones. This is because (1) fixed points reproduced better the exponential distributions of alpha fluctuations, (2) post-supercritical fixed points showed alpha power over the aperiodic component of the spectrum—as expected in resting-state—unlike pre-saddle node ones, and (3) the relationship between the increasing input to a node and the reduction in alpha power aligns with experimental data from fMRI-EEG co-registers reporting anti-correlations between alpha power and neuronal metabolism (Goldman et al., 2002; Laufs et al., 2003; Pang & Robinson, 2018). Across this range of fixed points, different levels of alpha power can be simulated. As the bifurcation parameter increases, the average alpha power decreases, potentially reaching a regime where no distinct alpha peak emerges above the aperiodic component.

It has been argued that the neural mass models used in whole-brain modeling might be parameterized at criticality, associating the critical points of a dynamical system with the criticality described in empirical neurophysiological data (Breakspear, 2017; Deco & Jirsa, 2012). At the critical point, the system would be able to switch between different modes of operation, providing flexibility and adaptability to the system. This idea has some support in the context of electrophysiological studies (Fontenele et al., 2019; Fuscà et al., 2023; Shew & Plenz, 2012), although still under discussion (Destexhe & Touboul, 2021). In either case, the extent to which the criticality in electrophysiology is well represented by the critical points of a dynamical system remains to be explored. In our data, we found two groups of single-node simulations showing bimodalities, and interestingly, both were parameterized at critical points of the system. However, in none of them the bimodality was due to biologically plausible transitions between modes of operation. In one of the groups, the bimodality was explained by the decaying initial transients of the system while, in the other, nodes were randomly switching between the pre-saddle fixed points and the limit cycle behavior of the JR, resulting in signals that do not resemble M/EEG recordings. We believe that a further understanding of criticality in electrophysiology and dynamical system is needed to better align simulated dynamics to empirical data.

This work advances the understanding of alpha brain activity by elucidating its dynamic properties and their implications for neural processing. Furthermore, it establishes a robust framework for assessing the biological plausibility of simulated neural dynamics, offering a systematic approach to bridging computational models with empirical data. By integrating theoretical insights with empirical validation, this framework enhances the reliability of simulations as tools for investigating brain function and dysfunction, while also offering new avenues to understand alpha rhythms and its involvement in cognition.

## Supplementary Material

Supplementary Material

## Data Availability

The dataset used in this study is available upon request. The code for analysis and simulations was written in Python and can be accessed at https://github.com/jescab01/AlphaFluctuations_2025.
