# Supplementary material for: The fluctuations of alpha power: Bimodalities, connectivity, and neural mass models
Source: Imaging Neurosci (Camb). 2025 Jul 2;3:IMAG.a.64. doi: 10.1162/IMAG.a.64 (PMC12330861; doi:10.1162/IMAG.a.64)
Supplement: Supplementary Material [file IMAG.a.64_supp.pdf]

Supporting Information

Figure S1

Intra-subject differences between rEC and rEO in the exponential modelling results and spectral variables. Values were averaged through subjects. A) Topological descriptions including the BIC difference between the unimodal and bimodal exponential fits (positive values in green indicate a tendency in rEC towards bimodality); the BIC value of the best fitted model per region (blue regions indicate a better fit in rEO than in rEC); and three spectral variables including IAF, alpha power and aperiodic exponent. B) Scatter plots with a dot per subject and region. The dashed line as 1:1 reference. Dots over the reference line represent increases in rEC.

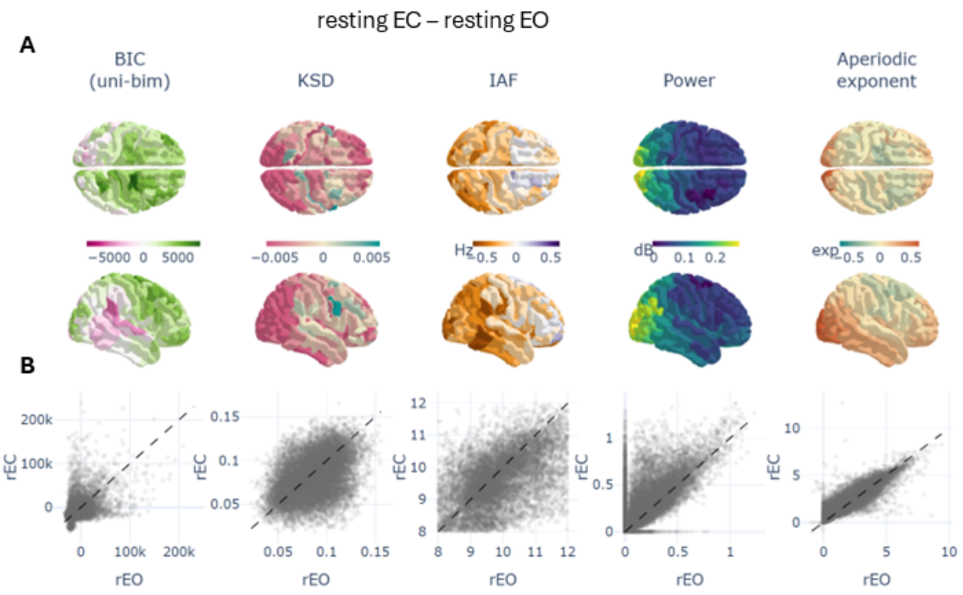

Figure S2

Boxplots showing the KSD results across subjects, conditions (rEC and rEO, color-coded), and brain regions, comparing empirical alpha power distributions to five theoretical models: exponential, Weibull, gamma, lognormal, and normal (Gaussian). All KSD tests were statistically significant (p ¡ 0.001), indicating that the empirical distributions differed significantly from their theoretical counterparts—likely due in part to the large sample size (180,000 data points per distribution). Among the models tested, the lognormal distribution yielded the best fit (KSD mean = 0.024, SD = 0.009), followed by the Weibull (KSD mean = 0.049, SD = 0.015), gamma (KSD mean = 0.052, SD = 0.024), and unimodal exponential (KSD mean = 0.079, SD = 0.030) distributions. As expected, the normal distribution showed the poorest fit (KSD mean = 0.175, SD = 0.042).

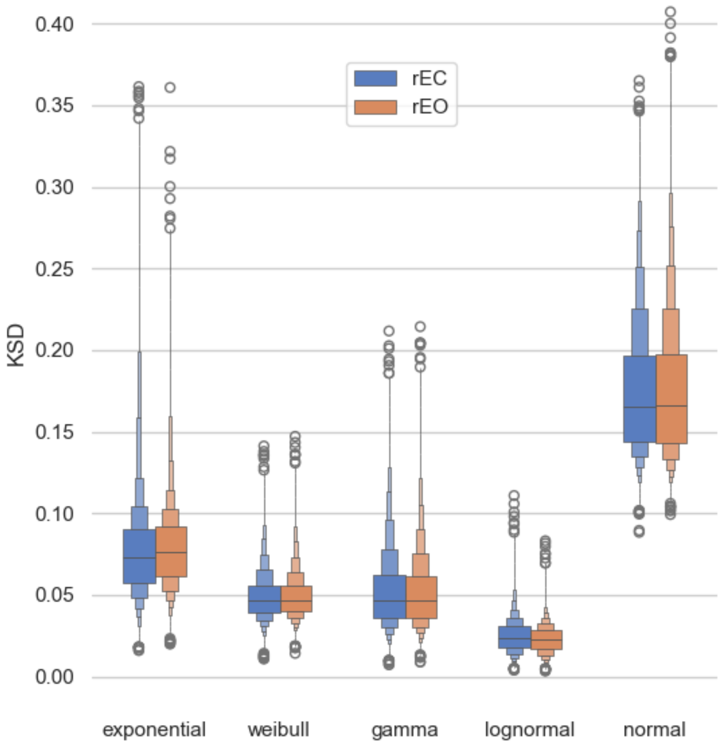

Figure S3

Correlations between alpha power and FC in different functional networks computed per frequency band (in rows) and averaging across connections (each datapoint represent one subject), differentiating by FC metric (ciPLV and cAEC in columns) and condition (rEC and rEO in sub-columns). In color, the different networks considered: default mode (DMN), visual (VIS), sensorimotor (SENS), dorsal attention (DAN), ventral attention (VAN) and salience (SAL) networks. A detailed list of the HCP regions included in each network is listed in supplementary Table S2. (*) corresponds to statistical significance after correction for multiple comparisons, with p-corr<0.01.

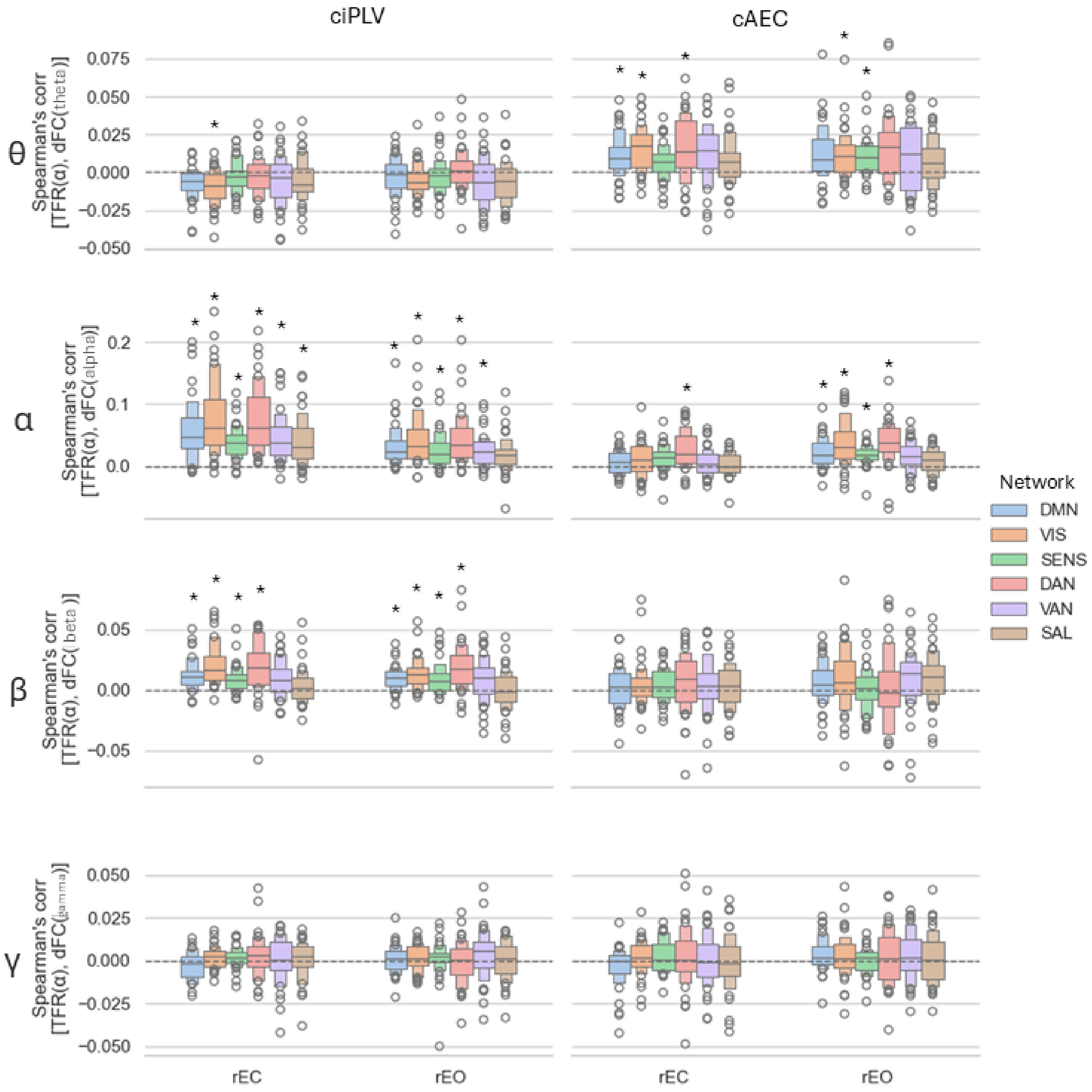

Figure S4

Samples of bimodalities found in single node simulations (Fig. ??) at the critical point of the subcritical bifurcation (p=[0.32, 0.325, 0.33]) with low noise (sigma = 0.00075). All the traces show a decaying initial transient that overcame the discarded initial 8 seconds of simulation. All the samples fitted better bimodal exponentials: green trace [BIC: unimodal = -83127.95; bimodal = -84328.11], orange trace [BIC: unimodal = -98014.40; bimodal = -99485.34], and blue trace [BIC: unimodal = -96800.84; bimodal =-100683.70]. Note how the initial transient becomes less evident as the model gets into the post-subcritical fixed point state.

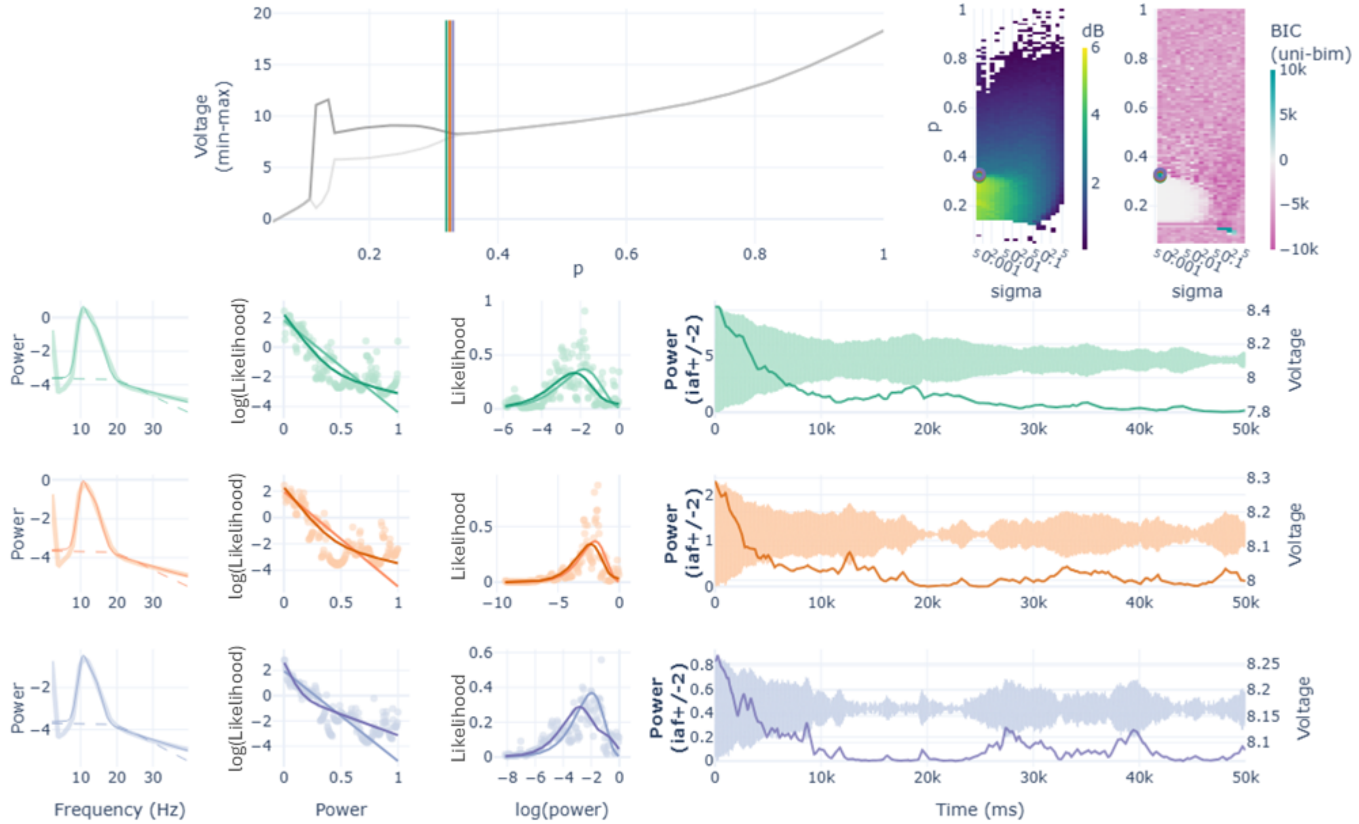

Figure S5

Same as in Fig 5.1 discarding longer transients (40 seconds): single node simulations at the critical point of the subcritical bifurcation (p=[0.32, 0.325, 0.33]) with low noise (sigma = 0.00075). With transients discarded bimodalities appeared less frequently. Here, all the samples fitted better unimodal exponentials: green trace [BIC: unimodal = -52965.25; bimodal = -39361.56], orange trace [BIC: unimodal = -86116.75; bimodal = -77302.37], and blue trace [BIC: unimodal = -65850.80; bimodal = -53257.19].

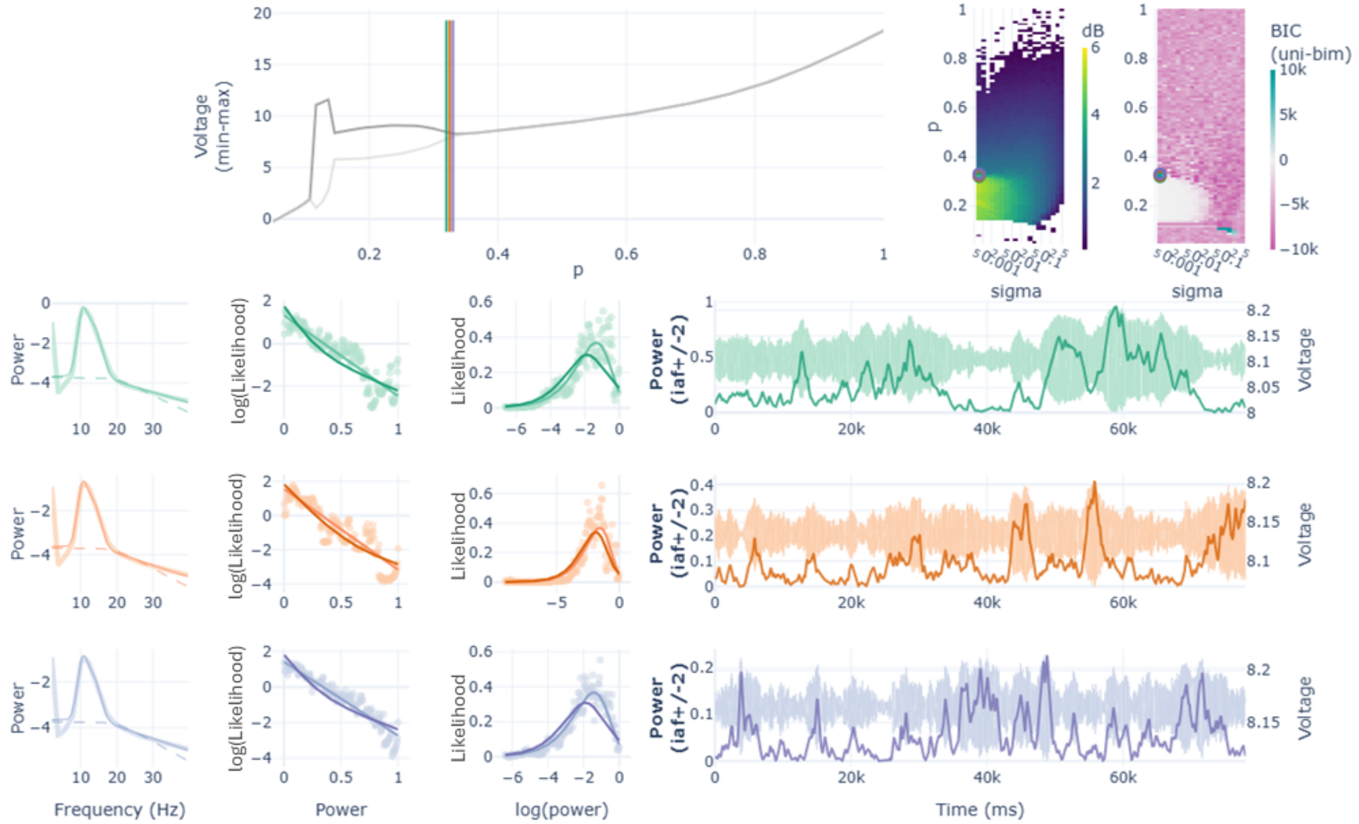

Figure S6

Samples of bimodalities found in single node simulations (Fig. **??**) at the saddle node bifurcation (p=[0.1025, 0.105, 0.1075] with high noise (sigma=0.1). These bimodalities represent the switching behaviour of the node between the fixed point state and the limit cycle. Note that increasing p, rises the frequency of switching. All samples fitted better bimodal exponentials: green trace [BIC: unimodal = -291663.23; bimodal = -304581.65], orange trace [BIC: unimodal = -224595.13; bimodal = -316788.16], and blue trace [BIC: unimodal = -151996.88; bimodal = -234628.44].

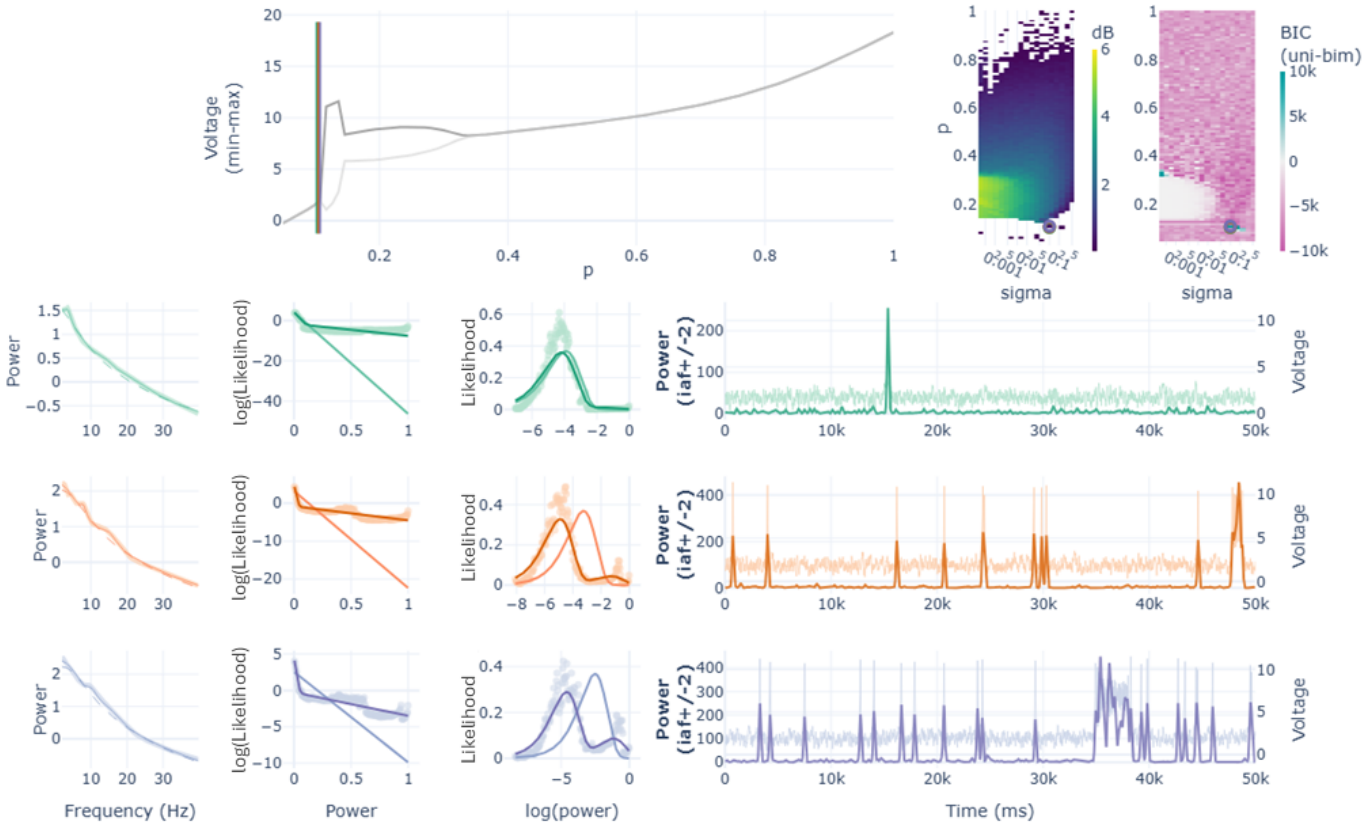

Figure S7

Samples of the network simulations for one region (V1 L). First row shows the bifurcation and two parameter spaces as reference for the picked simulations (in coloured vertical lines and open circles). Next rows show: 1) the power spectrum calculated from the data (thick line), the modeled spectra (thin line), and the modeled aperiodic component (dashed line); 2) the exponential function in two different coordinate spaces (i.e, Linear-Log and Log-Linear) with the scatter representing the histogram of the simulated TFR(α) and the lines representing both the unimodal (light colour) and the bimodal exponential models (dark colour); 3) the raw signal (light colour) and the TFR(α) values (dark colour) in time. The used parameters were (p, g) = [green (0.07, 20); orange (0.07, 80), blue (0.44, 20), pink (0.44, 80)].

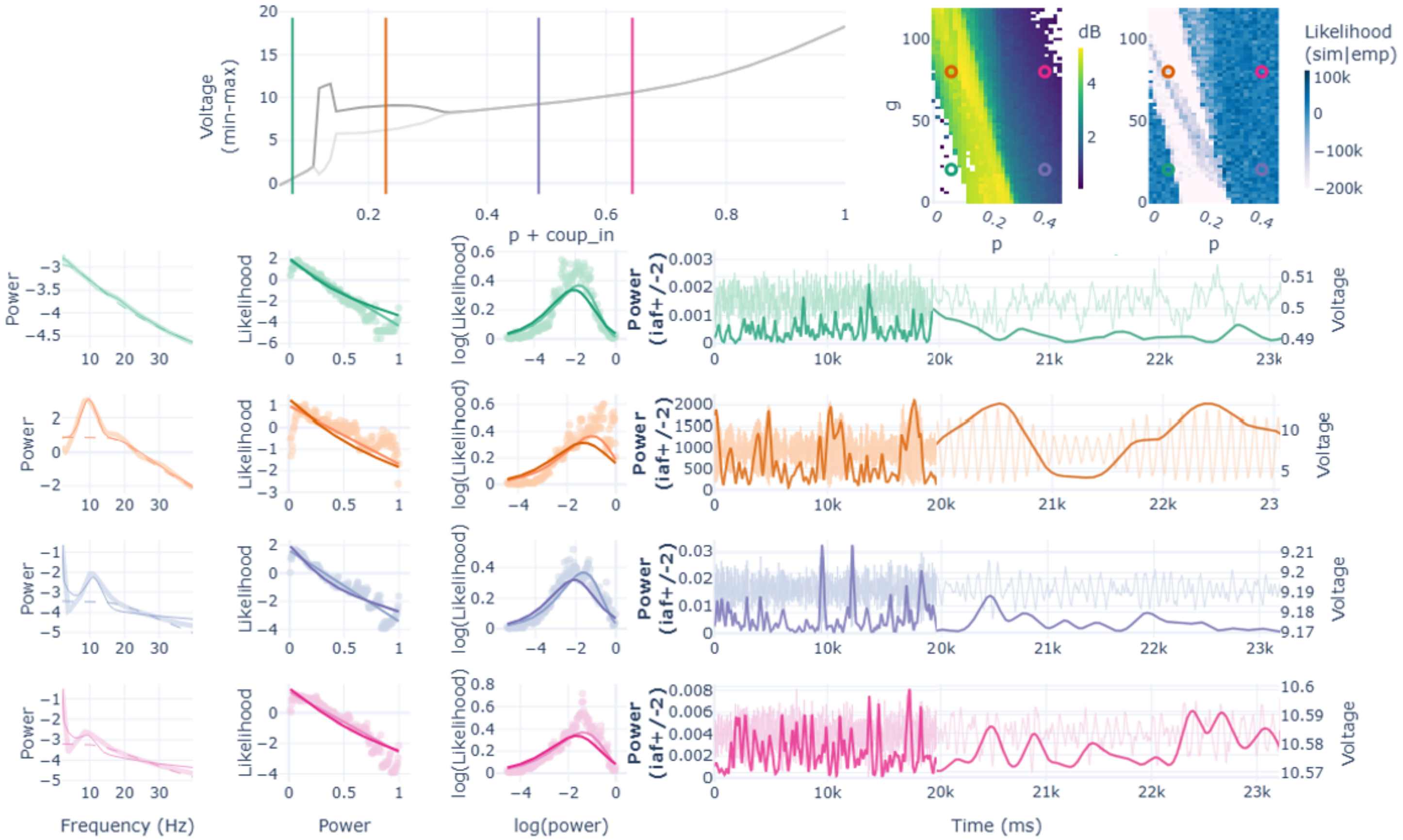

Table S1

Areas of the HCPex atlas and corresponding Region groups (Huang et al., 2021).

Area	Region
Primary Visual Cortex	Primary Visual Cortex
Second Visual Area	Early Visual Cortex
Third Visual Area	Early Visual Cortex
Fourth Visual Area	Early Visual Cortex
IntraParietal Sulcus Area 1	Dorsal Stream Visual Cortex
Area V3A	Dorsal Stream Visual Cortex
Area V3B	Dorsal Stream Visual Cortex
Sixth Visual Area	Dorsal Stream Visual Cortex
Area V6A	Dorsal Stream Visual Cortex
Seventh Visual Area	Dorsal Stream Visual Cortex
Fusiform Face Complex	Ventral Stream Visual Cortex
Posterior InferoTemporal Complex	Ventral Stream Visual Cortex
Eighth Visual Area	Ventral Stream Visual Cortex
VentroMedial Visual Area 1	Ventral Stream Visual Cortex
VentroMedial Visual Area 2	Ventral Stream Visual Cortex
VentroMedial Visual Area 3	Ventral Stream Visual Cortex
Ventral Visual Complex	Ventral Stream Visual Cortex
Area FST	MT+ Complex and Neighboring Visual Areas
Area Lateral Occipital 1	MT+ Complex and Neighboring Visual Areas
Area Lateral Occipital 2	MT+ Complex and Neighboring Visual Areas
Area Lateral Occipital 3	MT+ Complex and Neighboring Visual Areas
Medial Superior Temporal Area	MT+ Complex and Neighboring Visual Areas
Middle Temporal Area	MT+ Complex and Neighboring Visual Areas
Area PH	MT+ Complex and Neighboring Visual Areas
Area V3CD	MT+ Complex and Neighboring Visual Areas
Area V4t	MT+ Complex and Neighboring Visual Areas
Area 1	Primary Somatosensory Complex (S1)
Area 2	Primary Somatosensory Complex (S1)
Area 3a	Primary Somatosensory Complex (S1)
Primary Sensory Cortex	Primary Somatosensory Complex (S1)
Primary Motor Cortex	Primary Motor Cortex (M1)
Area 23c	Posterior Cingulate Cortex
Dorsal Area 24d	Cingulate motor areas
Ventral Area 24d	Cingulate motor areas
Area 5L	Area 5
Area 5m	Area 5
Area 5m ventral	Area 5
Area 6m anterior	Supplementary Motor Areas
Area 6mp	Supplementary Motor Areas
Supplementary and Cingulate Eye Field	Supplementary Motor Areas
Area 55b	Premotor Cortex
Area 6 anterior	Premotor Cortex
Dorsal area 6	Premotor Cortex
Rostral Area 6	Premotor Cortex
Ventral Area 6	Premotor Cortex
Frontal Eye Fields	Premotor Cortex
Premotor Eye Field	Premotor Cortex

Area 43	Posterior Opercular Cortex
Frontal Opercular Area 1	Posterior Opercular Cortex
Area OP1-SII	Posterior Opercular Cortex
Area OP2-3-VS	Posterior Opercular Cortex
Area OP4-PV	Posterior Opercular Cortex
Area 52	Insular and Frontal Opercular Cortex
Primary Auditory Cortex	Early Auditory Cortex
Lateral Belt Complex	Early Auditory Cortex
Medial Belt Complex	Early Auditory Cortex
ParaBelt Complex	Early Auditory Cortex
Area PFcm	Posterior Opercular Cortex
RetroInsular Cortex	Early Auditory Cortex
Auditory 4 Complex	Auditory Association Cortex
Auditory 5 Complex	Auditory Association Cortex
Area STGa	Auditory Association Cortex
Area STSd anterior	Auditory Association Cortex
Area STSd posterior	Auditory Association Cortex
Area STSv anterior	Auditory Association Cortex
Area STSv posterior	Auditory Association Cortex
Area TA2	Auditory Association Cortex
Anterior Agranular Insula Complex	Insular and Frontal Opercular Cortex
Anterior Ventral Insular Area	Insular and Frontal Opercular Cortex
Frontal Opercular Area 2	Insular and Frontal Opercular Cortex
Frontal Opercular Area 3	Insular and Frontal Opercular Cortex
Frontal Opercular Area 4	Insular and Frontal Opercular Cortex
Area Frontal Opercular 5	Insular and Frontal Opercular Cortex
Insular Granular Complex	Insular and Frontal Opercular Cortex
Middle Insular Area	Insular and Frontal Opercular Cortex
Para-Insular Area	Insular and Frontal Opercular Cortex
Pirform Cortex	Insular and Frontal Opercular Cortex
Area Posterior Insular 1	Insular and Frontal Opercular Cortex
Posterior Insular Area 2	Insular and Frontal Opercular Cortex
Hippocampus	Medial Temporal Cortex
PreSubiculum	Medial Temporal Cortex
Entorhinal Cortex	Medial Temporal Cortex
Perirhinal Ectorhinal Cortex	Medial Temporal Cortex
Area TF	Lateral Temporal Cortex and Temporal Pole
ParaHippocampal Area 1	Medial Temporal Cortex
ParaHippocampal Area 2	Medial Temporal Cortex
ParaHippocampal Area 3	Medial Temporal Cortex
Area PHT	Lateral Temporal Cortex and Temporal Pole
Area TE1 anterior	Lateral Temporal Cortex and Temporal Pole
Area TE1 Middle	Lateral Temporal Cortex and Temporal Pole
Area TE1 posterior	Lateral Temporal Cortex and Temporal Pole
Area TE2 anterior	Lateral Temporal Cortex and Temporal Pole
Area TE2 posterior	Lateral Temporal Cortex and Temporal Pole
Area TG dorsal	Lateral Temporal Cortex and Temporal Pole
Area TG Ventral	Lateral Temporal Cortex and Temporal Pole
PeriSylvian Language Area	Temporo-Parieto-Occipital Junction
Superior Temporal Visual Area	Temporo-Parieto-Occipital Junction
Area TemporoParietoOccipital Junction 1	Temporo-Parieto-Occipital Junction
Area TemporoParietoOccipital Junction 2	Temporo-Parieto-Occipital Junction

Area TemporoParietoOccipital Junction 3	Temporo-Parieto-Occipital Junction
Lateral Area 7A	Superior Parietal Cortex
Medial Area 7A	Superior Parietal Cortex
Area 7PC	Superior Parietal Cortex
Lateral Area 7P	Superior Parietal Cortex
Medial Area 7P	Superior Parietal Cortex
Anterior IntraParietal Area	Superior Parietal Cortex
Area Lateral IntraParietal dorsal	Superior Parietal Cortex
Area Lateral IntraParietal ventral	Superior Parietal Cortex
Medial IntraParietal Area	Superior Parietal Cortex
Ventral IntraParietal Complex	Superior Parietal Cortex
Area IntraParietal 0	Inferior Parietal Cortex
Area IntraParietal 1	Inferior Parietal Cortex
Area IntraParietal 2	Inferior Parietal Cortex
Area PF Complex	Inferior Parietal Cortex
Area PFm Complex	Inferior Parietal Cortex
Area PF Opercular	Inferior Parietal Cortex
Area PFt	Inferior Parietal Cortex
Area PGi	Inferior Parietal Cortex
Area PGp	Inferior Parietal Cortex
Area PGs	Inferior Parietal Cortex
Area 23d	Posterior Cingulate Cortex
Area 31a	Posterior Cingulate Cortex
Area 31pd	Posterior Cingulate Cortex
Area 31p ventral	Posterior Cingulate Cortex
Area 7m	Posterior Cingulate Cortex
Area dorsal 23 a+b	Posterior Cingulate Cortex
Dorsal Transitional Visual Area	Posterior Cingulate Cortex
PreCuneus Visual Area	Posterior Cingulate Cortex
Parieto-Occipital Sulcus Area 1	Posterior Cingulate Cortex
Parieto-Occipital Sulcus Area 2	Posterior Cingulate Cortex
ProStriate Area	Posterior Cingulate Cortex
RetroSplenial Complex	Posterior Cingulate Cortex
Area ventral 23 a+b	Posterior Cingulate Cortex
Area 10r	Medial Prefrontal Cortex
Area 10v	Medial Prefrontal Cortex
Area 25	Medial Prefrontal Cortex
Area 33 prime	Anterior Cingulate Cortex
Area 8BM	Medial Prefrontal Cortex
Area 9 Middle	Medial Prefrontal Cortex
Area a24	Anterior Cingulate Cortex
Anterior 24 prime	Anterior Cingulate Cortex
Area anterior 32 prime	Anterior Cingulate Cortex
Area dorsal 32	Anterior Cingulate Cortex
Area posterior 24	Anterior Cingulate Cortex
Area Posterior 24 prime	Anterior Cingulate Cortex
Area p32	Anterior Cingulate Cortex
Area p32 prime	Anterior Cingulate Cortex
Posterior OFC Complex	Orbital and Polar Frontal Cortex
Area s32	Anterior Cingulate Cortex
Area 10d	Orbital and Polar Frontal Cortex
Polar 10p	Orbital and Polar Frontal Cortex

Area 11l	Orbital and Polar Frontal Cortex
Area 13l	Orbital and Polar Frontal Cortex
Area 47m	Orbital and Polar Frontal Cortex
Area 47s	Orbital and Polar Frontal Cortex
Area anterior 10p	Orbital and Polar Frontal Cortex
Orbital Frontal Complex	Orbital and Polar Frontal Cortex
Area posterior 10p	Orbital and Polar Frontal Cortex
Area 44	Inferior Frontal Cortex
Area 45	Inferior Frontal Cortex
Area 47l (47 lateral)	Inferior Frontal Cortex
Area anterior 47r	Orbital and Polar Frontal Cortex
Area IFJa	Inferior Frontal Cortex
Area IFJp	Inferior Frontal Cortex
Area IFSa	Inferior Frontal Cortex
Area IFSp	Inferior Frontal Cortex
Area posterior 47r	Inferior Frontal Cortex
Area 46	DorsoLateral Prefontal Cortex
Area 8Ad	DorsoLateral Prefontal Cortex
Area 8Av	DorsoLateral Prefontal Cortex
Area 8B Lateral	DorsoLateral Prefontal Cortex
Area 8C	DorsoLateral Prefontal Cortex
Area 9-46d	DorsoLateral Prefontal Cortex
Area 9 anterior	DorsoLateral Prefontal Cortex
Area 9 Posterior	DorsoLateral Prefontal Cortex
Area anterior 9-46v	DorsoLateral Prefontal Cortex
Inferior 6-8 Transitional Area	DorsoLateral Prefontal Cortex
Area posterior 9-46v	DorsoLateral Prefontal Cortex
Superior 6-8 Transitional Area	DorsoLateral Prefontal Cortex
Superior Frontal Language Area	DorsoLateral Prefontal Cortex

Table S2

Brain functional networks based on the HCPex atlas (Huang et al., 2021) including the default mode (DMN) following Sandhu et al. (2020), visual following Glasser et al. (2016), sensorimotor following Glasser et al. (2016), salience following Briggs et al. (2022), dorsal attention (DAN) following Suo et al. (2021) and ventral attention (VAN) following Suo et al. (2021).

DMN Regions	Visual	Sensorimotor
Area 10r L	Primary Visual Cortex L	Area 1 L
Area 10r R	Second Visual Area L	Area 2 L
Area 31a L	Third Visual Area L	Area 3a L
Area 31a R	Fourth Visual Area L	Primary Sensory Cortex L
Area 31pd L	IntraParietal Sulcus Area 1 L	Primary Motor Cortex L
Area 31pd R	Area V3A L	Dorsal Area 24d L
Area 31p ventral L	Area V3B L	Ventral Area 24d L
Area 31p ventral R	Sixth Visual Area L	Area 5L L
Area a24 L	Area V6A L	Area 5m L
Area a24 R	Seventh Visual Area L	Area 5m ventral L
Area dorsal 23 a+b L	Fusiform Face Complex L	Area 6m anterior L
Area dorsal 23 a+b R	Posterior InferoTemporal complex L	Area 6mp L
Area IntraParietal 1 L	Eighth Visual Area L	Supplementary and Cingulate Eye Field L
Area IntraParietal 1 R	VentroMedial Visual Area 1 L	Area 55b L
Area p32 L	VentroMedial Visual Area 2 L	Area 6 anterior L
Area p32 R	VentroMedial Visual Area 3 L	Dorsal area 6 L
Parieto-Occipital Sulcus Area 1 L	Ventral Visual Complex L	Rostral Area 6 L
Parieto-Occipital Sulcus Area 1 R	Primary Visual Cortex R	Ventral Area 6 L
Parieto-Occipital Sulcus Area 2 L	Second Visual Area R	Frontal Eye Fields L
Parieto-Occipital Sulcus Area 2 R	Third Visual Area R	Premotor Eye Field L
RetroSplenial Complex L	Fourth Visual Area R	Area 43 L
RetroSplenial Complex R	IntraParietal Sulcus Area 1 R	Frontal Opercular Area 1 L
Area PFm Complex L	Area V3A R	Area OP1-SII L
Area PFm Complex R	Area V3B R	Area OP2-3-VS L
Area PGi L	Sixth Visual Area R	Area OP4-PV L
Area PGi R	Area V6A R	Area PFcm L
Area PGs L	Seventh Visual Area R	Area 1 R
Area PGs R	Fusiform Face Complex R	Area 2 R
Area s32 L	Posterior InferoTemporal complex R	Area 3a R
Area s32 R	Eighth Visual Area R	Primary Sensory Cortex R
Area TemporoParietoOccipital Junction 3 L	VentroMedial Visual Area 1 R	Primary Motor Cortex R
Area TemporoParietoOccipital Junction 3 R	VentroMedial Visual Area 2 R	Dorsal Area 24d R
Area ventral 23 a+b L	VentroMedial Visual Area 3 R	Ventral Area 24d R
Area ventral 23 a+b R	Ventral Visual Complex R	Area 5L R
Hippocampus L		Area 5m R
Hippocampus R		Area 5m ventral R
ParaHippocampal Area 1 L		Area 6m anterior R
ParaHippocampal Area 1 R		Area 6mp R
		Supplementary and Cingulate Eye Field R
		Area 55b R
		Area 6 anterior R
		Dorsal area 6 R
		Rostral Area 6 R
		Ventral Area 6 R
		Frontal Eye Fields R
		Premotor Eye Field R
		Area 43 R
		Frontal Opercular Area 1 R
		Area OP1-SII R
		Area OP2-3-VS R
		Area OP4-PV R
		Area PFcm R

**Salience**	**DAN**	**VAN**
Anterior Ventral Insular Area L	Frontal Eye Fields L	Anterior Agranular Insula Complex R
Anterior Ventral Insular Area R	Frontal Eye Fields R	Anterior Ventral Insular Area R
Middle Insular Area L	IntraParietal Sulcus Area 1 R	Area 44 R
Middle Insular Area R	Anterior IntraParietal Area R	Area TemporoParietoOccipital Junction 1 R
Frontal Opercular Area 4 L	Area IntraParietal 0 R	Area TemporoParietoOccipital Junction 2 R
Frontal Opercular Area 4 R	Area IntraParietal 1 R	Area TemporoParietoOccipital Junction 3 R
Area Frontal Opercular 5 L	Area IntraParietal 2 R	Anterior Agranular Insula Complex L
Area Frontal Opercular 5 R	Area Lateral IntraParietal dorsal R	Anterior Ventral Insular Area L
Anterior 24' L	Area Lateral IntraParietal ventral R	Area 44 L
Anterior 24' R	Medial IntraParietal Area R	
Area anterior 32' L	Ventral IntraParietal Complex R	
Area anterior 32' R	Lateral Area 7A L	
Area p32' L	Medial Area 7A L	
Area p32' R	Area 7PC L	
Supplementary and Cingulate Eye Field L	Lateral Area 7P L	
Supplementary and Cingulate Eye Field R	Medial Area 7P L	
Area 46 L		
Area 46 R		

References

Briggs, R. G., Young, I. M., Dadario, N. B., Fonseka, R. D., Hormovas, J., Allan, P., Larsen, M. L., Lin, Y., Tanglay, O., Maxwell, B. D., Conner, A. K., Stafford, J. F., Glenn, C. A., Teo, C., and Sughrue, M. E. (2022). Parcellation-based tractographic modeling of the salience network through meta-analysis. *Brain and Behavior*, 12(7).

Glasser, M. F., Coalson, T. S., Robinson, E. C., Hacker, C. D., Harwell, J., Yacoub, E., Ugurbil, K., Andersson, J., Beckmann, C. F., Jenkinson, M., Smith, S. M., and Van Essen, D. C. (2016). A multi-modal parcellation of human cerebral cortex. *Nature*, 536(7615):171–178.

Huang, C.-C., Rolls, E. T., Feng, J., and Lin, C.-P. (2021). An extended human connectome project multimodal parcellation atlas of the human cortex and subcortical areas. *Brain Structure and Function*, 227(3):763–778.

Sandhu, Z., Tanglay, O., Young, I. M., Briggs, R. G., Bai, M. Y., Larsen, M. L., Conner, A. K., Dhanaraj, V., Lin, Y., Hormovas, J., Fonseka, R. D., Glenn, C. A., and Sughrue, M. E. (2020). Parcellation-based anatomic modeling of the default mode network. *Brain and Behavior*, 11(2).

Suo, X., Ding, H., Li, X., Zhang, Y., Liang, M., Zhang, Y., Yu, C., and Qin, W. (2021). Anatomical and functional coupling between the dorsal and ventral attention networks. *NeuroImage*, 232:117868.
